# How to Hang Pictures

**Published:** 1881-07

**Authors:** 


### HOW TO HANG PICTURES.

NO picture ought to be hung higher than the height of the average human eye when the owner of the eye is standing. It is the most universal rule in our houses to hang pictures much above this level, and they cannot be enjoyed there. If the picture is a portrait, or it has human faces in it, its eyes should look as nearly into ours as possible; and if there be no such simple guide, perhaps a good rule will be to have the line that divides the picture horizontally into equal parts level with the eye. If one starts to hanging pictures with the determination to place them so that they can be easily seen and enjoyed without stretching the neck in the least, or stooping the body, he will be pretty sure to do well. In remote farm houses and country taverns we often see pictures, particularly portraits, skyed as high as if their owners had been academy hangers, and the painters young rivals of a new school. I suppose that the reason is that the simple-hearted owners think a picture such a precious thing that it cannot be hung too securely out of the reach of meddling hands. They are often not clear in their minds as to what the picture is meant for, and not finding it in any particular relation to human life and society they treat it with reverence, and put it where it will disturb them as little as possible. But, as people come to enjoy pictures and to get some intellectual, spiritual nourishment out of them, they want them as they want their books, where they can see them and use them.

